# The risk of corruption in public pharmaceutical procurement: how anti-corruption, transparency and accountability measures may reduce this risk

**DOI:** 10.1080/16549716.2019.1694745

**Published:** 2020-03-20

**Authors:** Jillian Clare Kohler, Deirdre Dimancesco

**Affiliations:** a Leslie Dan Faculty of Pharmacy, University of Toronto, Toronto, Ontario, Canada; b Essential Medicines & Health Products Department, World Health Organization (WHO), Geneva, Switzerland

**Keywords:** Anti-Corruption, Transparency and Accountability, Procurement practices, good governance, health, accountability, transparency, open contracting, e-procurement

## Abstract

**Background**: The goal of the public procurement of pharmaceuticals is to purchase sufficient quantities of high-quality pharmaceuticals at cost-effective prices for a given population. This goal can be undercut if corruption infiltrates the procurement process. Good procurement practices can help mitigate the risks of corruption and support equitable access to affordable and high-quality medicines.

**Objectives**: This paper aims to 1) examine manifestations of corruption in the pharmaceutical procurement process and key factors behind them, and 2) identify how to design and implement effective anti-corruption, transparency and accountability mechanisms within this process.

**Methods**: This paper was informed by a narrative literature review from 1996 to the present. The search focused on publications that addressed the issue of pharmaceutical procurement and governance and corruption issues. Our search included peer-reviewed literature, books, grey literature such as working papers, reports published by international organizations and donor agencies, and some media articles. Some documents used in this paper were already known to the authors.

**Results**: Procurement is highly vulnerable to corruption particularly in the health sector. What is more, corruption in the procurement process does not appear to be limited to any one level of government or type of health system. The better integration of accountability, transparency and anti-corruption mechanisms in the procurement process is needed to reduce the risk of corruption.

**Conclusions**: Lessons learned suggest that anti-corruption, transparency and accountability mechanisms in the pharmaceutical procurement process, such as open contracting and integrity pacts are helpful towards reducing the risk of corruption.

## Background

Public procurement is the purchase by public authorities of goods, services, and works. Governments are responsible for ensuring that procurement is carried out efficiently and with due diligence to guarantee quality of public service delivery and to protect the public interest [1]. In OECD countries, as much as 12 percent of GDP is spent on public procurement; health procurement is the second largest spending category [1,2]. As we seek to advance global access to medicines and other essential goods, we need to consider what are priority challenges to address. This includes corruption; public procurement holds the greatest risk for corruption amongst all government functions [3]. While the conditions which lead to corruption within the procurement process are many, what is certainly a facilitator is the potential for substantial financial rewards from large procurement contracts [4].

Despite its nebulous nature, corruption is a well-known threat to development. It is global in reach and manifests itself in different forms and all types of organizations [5]. Transparency International defines corruption as, ‘the misuse of entrusted power for private gain’ [6,7]. Klitgaard, on the other hand, describes it in part as ‘the monopoly of power combined with discretion and absence of accountability’ [8]. Corruption fuels inequity as it skews how resources are distributed and creates barriers to public health services and goods. Corruption can allow for the wealthy to accumulate more wealth while the most disadvantaged suffer. In short, ‘corruption hurts health outcomes and it is the poor who are affected the most’ [9].

Corruption is not limited to a particular type of health system structure. It is present in systems that are public or private, well or poorly funded, and technically simple or complex [10]. More often than not, corruption is difficult to verify because it is often hidden. It may also be challenging to identify if an act is corrupt or an example of administrative inefficiency. Equally important, even if corruption is suspected, its reporting may be limited due to fear of reprisals.

The risk of corruption in public procurement in particular is well known globally [3]. Indeed, Article 9 of the United Nations Convention Against Corruption calls for governments to ensure an appropriate system of public procurement [11]. Despite global anti-corruption efforts to date, public procurement contracts remain highly vulnerable to corruption [11]. Even though corruption is a real and clear danger to achieving goals such as better access of the population to medicines, given its complexity, we still need to learn more about what interventions can work best to reduce its risk, when and where. This lack of knowledge undeniably creates challenges to the design and implementation of anti-corruption initiatives [12]. Careful attention is therefore needed to determine what anti-corruption, transparency and accountability (ACTA) measures can best reduce corruption in the health sector and ensure that health goals are not compromised by it.

In the ideal case, good procurement of pharmaceuticals supports access to medicines for the population and contributes to Sustainable Development Goal 3: Good Health and Well-Being [13]. When corruption infiltrates the pharmaceutical procurement processes, public health goals are undercut by such results as pharmaceutical shortages, inflated drug prices, and the distribution of falsified and substandard medicines. If resources allocated to pharmaceutical procurement is wasted due to corruption and operational deficiencies, not only the pharmaceutical system is affected; the health system as a whole loses out on effectiveness and equity. Of equal importance, the public may very well lose trust in the government to uphold its social contract [14].

A common challenge for many countries is how to reduce the risk of corruption in the procurement of medicines [15]. Publicly funded medicines are particularly vulnerable to corruption if there is not sufficient documentation of the processes and there is weak governance [16]. The health system as a whole may become less effective and equitable [14]. Within the pharmaceutical system, corruption creates barriers to accessing medicines for those who are most vulnerable. The United Nations has notably recognized the serious threat corruption presents to the realisation of human rights [17].

The availability and affordability of pharmaceutical products is certainly affected by the efficiency and governance of the procurement process [18]. To be sure, many low- and middle-income countries are often contending with sub-optimal public supply systems. This may be the result of factors such as overall neglect, poor management, a lack of transparency, and not enough qualified procurement specialists [19]. These subpar conditions will also create opportunities for corruption to emerge, which likely will put a dent into any effort to advance equitable access of the population to essential medicines.

One of the reasons why corruption in procurement systems may persist is that even when procurement reforms take place, often broader ACTA measures are not integrated into them. We thus advance the argument that it is not only important but essential to integrate ACTA measures into pharmaceutical procurement practices if we hope to ensure better access of the population to essential medicines. The timing is opportune, inasmuch as WHO is putting more focus on addressing corruption-free procurement during its programming for 2019–2023 [20].

## Objectives

The objectives of this paper are twofold: to examine manifestations of corruption in the pharmaceutical procurement process and key factors behind them; and to discuss what ACTA mechanisms can help reduce the risk of corruption in the pharmaceutical procurement process. This paper contributes to the very crowded literature space on pharmaceutical procurement by focusing on how corruption is a risk to corruption within the space of pharmaceutical sector procurement and what ACTA measures may reduce its risk.

## Methods

This paper was informed by a narrative literature review that identified literature from 1996 to the present. More narrowly, our search focused on publications that addressed the issue of pharmaceutical procurement and governance and corruption issues. Our search included peer-reviewed literature, books, grey literature such as working papers, reports published by international organizations and donor agencies, and some media articles. Some documents used in this paper were known to the authors and identified prior to this search.

The Search Engines PubMed, PAIS, and Summon were used to identify key literature using the keywords ‘corruption AND procurement,’ ‘corruption AND procurement AND (pharmaceuticals or medicine),’ ‘corruption AND pharmaceutical AND system,’ ‘procurement AND pharmaceuticals AND governance’ ‘corruption in procurement,’ ‘corruption in pharmaceutical system,’ ‘corruption in procurement medicines,’ and ‘pharmaceuticals and governance.’ These searches first generated 2,038 results; 1,938 titles were then eliminated. Duplicate articles were removed, and articles were filtered so that only those that focused on governance issues in the pharmaceutical procurement process and/or ACTA measures in pharmaceutical procurement remained. This resulted in 99 articles which then was reduced to a final pool of 62 articles that were cited in this paper. The literature search was limited insofar as only English sources were used and the articles cited in this paper are not exhaustive.

Our paper is organized in the following way: first, we discuss key factors, types, and examples of corruption in pharmaceutical procurement; second, we explain how transparency and accountability can help reduce corruption; and third, we illuminate examples of ACTA mechanisms that have shown to help reduce the risk of corruption in procurement.

## Results

### How corruption can manifest itself in pharmaceutical procurement drivers

Klitgaard (1988) explains that there are certain conditions which are a prerequisite for corruption to take place: opportunities to make a profit, a general lack of accountability, and individuals with discretionary powers [8]. These conditions may be present in the procurement process. For instance, when there are circumstances with limited or no transparency and accountability, profit may be extracted by an official inflating a contract’s value or manipulating contract stipulations. Quiet corruption may also take place, such as when the public and/or government officials fail to question cost overruns [21].

### Types

When designing ACTA mechanisms, it is critical to identify what type of corruption is being targeted. Corruption can emerge in many forms but for the purpose of our focus on pharmaceutical procurement, we highlight two that may be found in the procurement process: Isolated corruption consists of a very limited number of individuals and acts, and thus relatively straightforward to investigate and sanction, although seldom detected. This may include improper gifts and bribes given to a procurement official working on a tender of low value [22]. Systemic corruption, on the other hand, is an entrenched phenomenon in dealings between the public and private sector, widely accepted as routine, and thus on a much larger scale [23]. This may take the form of extensive, complex systems that are difficult to untangle [22] and also involve the participation of organized crime [24].

### Manifestations

Throughout the procurement tendering process, namely pre-bidding, bidding and post-bidding, there are a number of clear corruption risks. These are highlighted in Table 1. Some examples include ‘different’ bidders having the same contact information, forged documents, colluding companies, and bribes and kickbacks to government officials [25].

**Table 1.** Corruption risks and ACTA interventions in pharmaceutical procurement.

Stage in procurement process	Manifestations	Examples of acta interventions
Pre-bidding	Falsified type or amount of product Fabricated bidders Bids drafted to favour a particular company Forged documentation Bidding vendors provide bribes and kickbacks to government officials Information regarding contracts distributed in an unequal manner	Giving all eligible bidders the opportunity to participate Enhancing transparency in bidding process Publicizing tender criteria Putting in place integrity pacts Procurement agency issues clear and transparent policies and procedures that are publicly available. Training of procurement officers on policies and procedures as well as how to detect potential corruption within the procurement process. Providing clear policies and procedures for the national procurement regulatory agency. Regular checks on procurement processes and outcomes by outside watchdog agency
Bidding	Tender influenced by bribery and extortion Conflicts of interest that may influence tenders overlooked Exclusion of bids not justified	Ensuring transparent and open bidding process through mechanisms such as electronic bidding Creating conflict of interest policies with appropriate measures to manage them Ensure national public procurement agency monitors the implementation of procurement rules by procuring entities (such as Ministry of Health). Introduction of a formal appeals process
Post-Bidding	Falsified invoices Inflated contracts Rewritten contract terms Goods not delivered	Publicizing information about bid chosen and rationale Disclosing bids that did not win Citizen monitoring of contract execution Evaluating company performance Conducting formal audits

Based on [15,26,27].

The pre-bidding stage involves a needs assessment, definition of contract characteristics, and the selection of a procurement method. Corruption may happen when a need is falsified, or a bid is drafted to favour a predetermined company; the latter often suggests that some kind of kickback was offered to one or more government officials. The bidding stage involves bid invitations and evaluations and the contract award. This stage is vulnerable to direct procurement corruption when a tender winner is chosen through bribery or extortion. In the post-bidding stage, contract implementation and monitoring are the main activities. Corruption in these instances may include false invoicing and rewriting of the terms of the contract agreements.

### Where corruption in public procurement occurs

Corruption in procurement is not limited to one location or type of procurement system. It can occur at the national, state/provincial, or local levels, and is found in centralized and decentralized procurement systems alike. Decentralization can potentially curb the risk of corruption if constituents have more access to more information about the procurement process and thus hold public officials accountable for procurement outcomes [28]. However, decentralization is not a solution, as if there are weak transparency and accountability mechanisms throughout the procurement process, corruption may still result [3].

### Examples

The World Bank’s Detailed Implementation Review of India (FY 2007–2008) is a clear example of how corruption in procurement can undercut intended development goals. The World Bank’s Review included a broad-based forensic review of procurement practices in five World Bank health projects in the country. The review discovered evidence of fraud and corruption in all of the projects, undercutting their goals [29]. The corruption included collusive behaviours, bribery, manipulated bid prices, deficient civil works certified as complete, broken or damaged equipment certified as compliant with specifications, under delivery of services, and inadequate project audit and control systems [9]. As a result of these findings, the World Bank initiated anti-corruption interventions in the projects, including procurement audits, community oversight and monitoring, social audits, web publication of all procurement processes, bidding and holding wrongdoers accountable. Importantly, sanctions were placed on those companies that breached the law. For example, two companies found guilty of collusive behaviour under the Reproductive and Child Health Project I in India were barred from participating in World Bank procurement tenders for a set period of time, although their complete barring from tenders would likely have been a more appropriate in terms of signaling that corrupt acts can result in heavy penalties.

Corruption in the procurement process can also include private financial interests influences what medicines are procured for the health system. It may manifest itself as kickbacks or bribes to bidders by suppliers in order to gain access to confidential information. It can also include direct procurement instead of competitive bidding without sound justification [30]. In a well-cited study of public hospitals in Latin America, using hospital records on a selection of similar supplies, it was found that the prices paid for supplies varied significantly across hospitals in all countries studied. The studies compared the ratios of high-to-low purchase prices and found that they varied from 3:1 to as much as 35:1. Discrepancies were explained by either mismanagement or corruption [31]. Fraud in procurement is a particular risk in hospitals, because almost all capital expenses involve procurement and often technical expertise is limited. To take but one example, doctors may request specific products and equipment due to payoffs [32].

A 2016 Transparency International report illuminates how in one country a coalition of civil society organisations (CSOs) discovered that shell companies were being used to manipulate market prices. Even though over 200 companies were licensed to provide pharmaceuticals, 83 percent had never won a public contract and many only existed on paper. In reality companies controlled 88 percent of the market. The CSOs learned that the then health minister had used state-of-emergency rules to purchase more than half of the essential medicines from a single supplier. Also, the prices paid for these medicines were as much as 41 percent higher than the international averages [33].

### How components of good governance – transparency and accountability – may help reduce corruption in pharmaceutical procurement

One entry point through which corruption is often addressed is through the conceptual framework of good governance. This framework has a variety of meanings. Many are based on the mechanisms through which it can be achieved, such as democracy, transparency, efficient public services, and the presence and enforcement of civil rights. Other definitions are based on specific outcomes, such as assuring that the most marginalized groups in society have a voice and receive fair, equitable treatment. The United Nations Economic and Social Commission for Asia and the Pacific (UNESCAP) defines good governance as ‘the exercise of economic, political and administrative authority to manage a country’s affairs at all levels, comprising the mechanisms, processes, and institutions through which that authority is directed.’ UNESCAP proposes eight characteristics of good governance: ‘It is participatory, consensus oriented, accountable, transparent, responsive, effective and efficient, equitable and inclusive and follows the rule of law’ [34].

Good governance generally requires investment (in other words, a commitment to transparency and accountability) from key stakeholders such as the private sector, civil society, and the state and is a prerequisite for sustainable human development [35,36]. It is viewed as essential for advancing efficient and effective health-care systems and for mitigating mismanagement [37]. The WHO regards it as a key building block of the health system, helping ensure that adequate policies, effective oversight, and strong accountability mechanisms are in place for the system’s proper design and management [35,36].

Thus, the complexities of the health system (in this instance, how pharmaceuticals are procured) require tailored governance approaches to identify vulnerabilities to corruption, waste, mismanagement, and fraud [38]. Anti-corruption efforts that embed good governance principles, this includes the integration of ACTA mechanisms, are considered to be the standard for tackling sector vulnerabilities [12].

### Transparency

Governing institutions are most able to meet the needs of a community when their processes include the good governance components of transparency and accountability. Transparency is ‘the degree to which access to government information is available’ [38–39]. ‘Understanding how decisions are made requires information about the procedures followed and the criteria used by policy makers to reach decisions’ [42–44]. ‘Understanding why decisions are made necessitates disclosure of the information drawn on by policy makers and revelation of the arguments adduced in favour and against particular decisions’ [45].

Greater transparency in the pharmaceutical procurement process allows for easy comparison of prices paid by different facilities for the same medicine in a decentralized system. This allows health-care facilities to make more informed decisions and can, over time, lead to greater purchasing power to negotiate prices with suppliers. Ideally, these measures help curb price gouging, price manipulation, and overpayments. Importantly, data can illuminate patterns and any outliers, which may suggest that there are overpayments, collusion, or kickbacks happening in the procurement process. Ultimately, transparency can be understood as governments making information publicly available so that their actions and decisions are visible and understandable to the public, who can in turn hold them accountable [43].

### Accountability

Accountability is the ‘mechanism that make individuals or agencies answerable or responsive to their particular publics’ [39]. Vian and Kohler (2016) point out that accountability makes reference to the mechanisms that make institutions responsive to the publics they serve [41,44,46]. They write that accountability matters because it helps to ensure that relevant entities answer to those who will be affected by decisions or actions taken by them. Accountability can reduce corruption and other abuses, assure compliance with standards and procedures, and improve performance and organizational learning. It also demands that institutions explain and justify their results to internal and external monitors or stakeholders and impose sanctions when performance falls short or corruption is found. Described differently, accountability is when ‘A is accountable to B when A is obliged to inform B about A’s (past or future) actions and decisions, to justify them, and to suffer punishment in the case of eventual misconduct’ [47].

### Gaining policy traction

Good governance has thus become an accepted conceptual framework for international institutions and their member governments to use in their efforts to promote accountability and transparency and possibly also to use good governance as a way to deter corruption. The assumption here is that the risk of corruption is reduced or even avoided if international institutions are accountable and transparent in their procurement practices and more broadly [48]. In this context, the WHO has promoted transparency and accountability through its Good Governance for Medicines Programme and with support of the WHO Collaborating Center for Governance, Accountability and Transparency in the Pharmaceutical Sector. There is indeed a growing consensus that the higher the levels of accountability and transparency, the more likely corruption and/or inefficiencies will be reduced [49].

For instance, transparency is Principle 1 of the OECD Principles for Enhancing Integrity in Public Procurement: ‘Provide an adequate degree of transparency in the entire procurement cycle in order to promote fair and equitable treatment for potential suppliers’ [50, p. 11]. Pursuant to the May 2016 Anti-Corruption Summit, the World Bank agreed on enhancing transparency as a key item to follow up on.

Another example is the G20 Principles for Promoting Integrity in Public Procurement include the call for ‘transparency of public procurement opportunities and awards, except where reasonable exceptions apply (e.g. security concerns or low-value procurements)’ [51]. As a follow-up to these principles, the G20 Anti-Corruption Implementation Plan for 2017–2018 underscores that G20 countries will ‘promote transparency in public contracting, including the use of open data across the contracting cycle, consistent with applicable law, and the use of e-procurement’ [52].

Trying to ensure the effective implementation of mechanisms to support good governance is not without its challenges. For example, Kohler and Ovtcharenko (2013) conducted a study on good governance for medicines initiatives led by international organizations, such as the World Bank and the WHO. The authors reported that these organizations encountered many obstacles notably linked to cultural and behavioural factors, resistance to change, lack of political support and commitment, and insufficient resources. The study suggests that while more focused good governance efforts that target a specific area could be useful, they alone may not result in the systematic changes necessary to improve the overall performance of the health system [53].

### Examples of ACTA mechanisms in procurement

While organizations such as the Global Fund and the UNDP advance a policy of ‘zero tolerance’ for corruption, its complete eradication is likely aspirational. So, it seems more realistic to focus on how the risk of corruption can be reduced (as opposed to eliminated) through the application of the context-specific ACTA policy and technology tools. First, policy measures include the advancement of transparency, non-discrimination, equality of access, open competition, accountability, value for money evaluation, related policy compliance [25], and a well-vetted expert committee with sufficient resources and power to undertake internal and external audits [54]. The onus should be on the committee to clarify their decision-making processes through written guidelines and Standard Operating Procedures (SOPs) that can be referred to in order to hold individuals accountable in case corrupt behaviour is detected [55]. This should be done frequently and over a prolonged period of time – particularly near the end of a fiscal year when there is an increased risk of corruption. Conflict-of-interest declarations can also be used to monitor relationships between physicians and the pharmaceutical industry and to avoid pre-bidding corruption, so long as there are adequate follow-up measures to address conflict of interest once it is revealed [22].

Technological tools such as e-procurement as discussed by Mackey and Cuomo in this volume may too help reduce corruption risks in procurement. Electronic bidding can help disseminate information about procurement procedures and results and encourage price competition. Other options include capping the maximum price of pharmaceuticals, prequalifying suppliers to ensure they are reliable, establishing a product defect reporting system, and providing constant technical assistance and training to procurement officers to ensure that procurement is carried out based on evidence and technical knowledge [9,55]. Accountability can be further advanced by ensuring procurement performance indicators as well as the price of supplies are regularly updated and analyzed.

The case of Ukraine shows the benefits of using technology in public contracting, inasmuch as it helped create a more competitive, transparent and accountable procurement system. Ukraine supported its procurement practices first by passing a law that supports transparency and promotes fair competition in the public procurement systems [56]. This resulted in a reformed procurement process that resulted in a centralized e-procurement system that is known as ProZorro. ProZorro is based on three principles: a hybrid open-source electronic system; ‘Everyone can see everything;’ and a Golden Triangle partnership. The latter refers to a collaboration whereby the government establishes the rules and protects information, the private sector provides the platform interfaces for contracting authorities and suppliers and civil society monitors the procurement process to ensure it is in line with the law. Prozorro also uses the Open Data Standard, promoting information access in the procurement process, and creating equal opportunities for companies to participate in public tenders [57].

Another example of how transparency improved procurement processes is found in the case of Chile. Chile’s pharmaceutical procurement system CENABAST (operating as a Central Medical Store) had historically been inefficient and non-transparent. Key changes put in place included the transition to an electronic bidding system and active use of the Internet to disseminate information about the procurement process publicly. The increased transparency of the pharmaceutical procurement process helped foster a critical mass of suppliers to compete for each product being tendered. This resulted in price competition and also served as an anti-corruption tool [58]. A more recent report from CENABAST confirmed efforts are still being made to improve health procurement processes in Chile. In 2013 their efforts reportedly increased transparency in 180 hospitals [59].

### The potential benefits of open contracting and e-procurement for advancing transparency and accountability

Open contracting is growing in popularity amongst governments and other purchasers as a mechanism for promoting transparency and accountability. Open contracting is a way to identify and fix problems within government contracts by engaging those outsides of government such as citizens and the private sector; this is done by publishing information on government contracts and ensuring this information is available to the public [60]. It is designed to ensure better value for money spent for governments, increase competition, deliver better quality goods to citizens, and reducing the risk of fraud and corruption [60]. In addition, open contracting is supported by the Open Contracting Data Standard, an international standard that helps promote transparency and accountability in the procurement process which includes planning, tender, award, contract and implementation [61]. It does this through the disclosure of data at all phases of the contracting processes by defining a common data model. The standard was established in order to improve transparency in the contracting process and to allow for a more comprehensive analysis of contracting data by more users [60]. It advances the creation of accessible, responsive information by allowing for the analysis of who is buying what, from whom, and at what price. Of note, it also provides information on bids and bidders who did not win the contract.

Open contracting, however, is a necessary but insufficient condition for reducing corruption in the procurement process. It needs to be accompanied by additional measures that promote good governance. For example, implementing integrity pacts, in tandem with open contracting can help ensure that corruption in procurement processes is less likely. These have been applied not only in the health sector but in other sectors, such as education and transportation. These pacts have been described as an ‘agreement between a government or government agency and all bidders for a public sector contract, which sets out rights and obligations to the effect that neither party will engage in corrupt conduct’ [33]. The pact demands that all parties will agree not to engage in bribery, collusion, or any other corrupt practices for the extent of the contract. Of equal importance, to ensure that the pact is upheld, a monitoring system, usually managed by civil society groups, is put in place [7].

It is also important in open contracting to ensure that when red flags appear, indicating a potential breach of standards, to ensure that the red flag is investigated to determine whether or not corruption is a factor. Red flags are triggered by using procurement data to detect corruption risk through the use of an algorithm. Further information on different digital technologies in different stages of development used to enhance transparency in medicines procurement (including additional discussion about e-procurement systems and open contracting), accompanied with discussion about technical challenges and opportunities faced by these digital tools, is provided in a separate study by Mackey & Cuomo in this volume.

What is also a promising technology in procurement is the use of electronic bidding, that ideally creates a platform through which multiple health-care facilities can upload tenders and prequalified suppliers (reliable suppliers that a government agency has preapproved as capable of delivering the particular products) can participate. If designed appropriately, open contracting in tandem with e-procurement helps increase the transparency and accountability of procurement procedures and prices by allowing the collection of data on tender bids, tender offers, terms and conditions, contract awards, supplier performance, and prices paid that can be disseminated to all health-care facilities and the public. E-procurement can also reduce supply costs, save time, and increase compliance with supplier contracts. In short, it is potentially efficient and transparent. Most of the challenges identified with e-procurement have been linked to information, communication, and technology issues [62].

**Box 1.** Uganda and electronic procurement.

In 2017, Uganda was one of the early adopters in Sub-Saharan Africa to move to an electronic procurement system that integrates the Open Contracting Data Standard (OCDS). This means that procurement data (for all publicly purchased goods) are in a format that is shareable, machine readable and ultimately easily accessible and usable for a broad audience (governments, CSOs and the private sector). Therefore, data can be used to inform decision- making, monitor procurements, and flag instances of misuse of public funds. Procurement entities at all government levels are trained by the Uganda Public Procurement and Disposal of Public Assets Authority (PPDA) to use the new system which is also regularly updated to increase efficiency and accuracy of the data collected. The OC4H has recently run a pilot in the Lira District, in the Northern region of Uganda. The pilot aimed to better understand the challenges and opportunities of introducing OCDS at the sub-national levels. Overall the findings revealed a familiarity with OCDS among procurement officials, despite room for improvement on the more technical aspects. However, it became clear that a focus on OCDS implementation may neglect other important aspects that impact on how data are used and analysed – in particular a strong understanding of how procurement works (including the regulatory framework) and the ability to use the data in a way that meet specific needs (e.g. improving procurement monitoring, increasing value for money, and/or tackling corruption). To ensure that open contracting and the introduction of OCDS do not remain a mere data input exercise, it is important to discuss open contracting and its benefits with those that are tasked with making decisions on procurement and on inputting data in the electronic Government Portal on a daily basis. Strengthening the capacity of data users and creating a need for open contracting are necessary to achieve change.Source [7]

**Box 2.** The Global Fund’s Wambo.

The use of online tools is becoming increasingly more common in procurement processes in an effort to enhance transparency and help improve competitive pricing. For example, Wambo.org is an online procurement tool that was launched in February 2016 by the Global Fund. Its aims to provide better market visibility and choice for health products that bought through catalogue purchases. It is available to Fund recipients who can use it to search, compare, purchase, and track the delivery of health products. Wambo has two types of memberships for its users: (1) those who use it to search and compare product prices; and, (2) those who use it to buy from catalogues and Long-Term Agremments. Orders, once confirmed, are billed directly to the recipient’s grant. In short, Wambo aims to increase transparency and help ensure the consistent supply of medicines, health products, and non-health commodities for programming under the Fund.Source: [62]

## Conclusion

This article highlights findings from our narrative literature review that focused on corruption risks in the public procurement of pharmaceuticals and ACTA measures to address it. The intent of this article was to strategically illuminate key issues and to provide examples related to the topic. Corruption significantly undercuts public health goals, including ensuring access to essential medicine, which is a sub-target of Sustainable Development Goal # 3. Our review illuminates how corruption in procurement often occurs when there are insufficient ACTA measures embedded throughout the procurement process from pre-bidding to delivery of goods. Evidence suggests that procurement can be strengthened through the integration of ACTA in its processes. This may include first undertaking corruption risk assessments in the pharmaceutical procurement area and based on findings from the assessments implement appropriate ACTA measures. Equally important, risk management strategies to address corruption, which is discussed by others in this volume, are helpful to apply in the procurement of medicines, in order to ensure that if there are potential vulnerabilities to corruption, they are identified and appropriately addressed. Still, knowing what is needed to be put in place to ensure the sustainability of ACTA measures procurement, does demand further research and capacity building, and could potentially be an area of focus for the WHO, the UNDP and the Global Fund under the ACTA for Health Alliance.

## Paper context

It is well documented that public procurement generally and in the health sector, in particular, is vulnerable to corruption. When corruption infiltrates the pharmaceutical procurement process, it is a threat to ensuring access of the population to essential medicines. This paper illuminates how procurement reforms should vitally include anti-corruption, transparency, and accountability (ACTA) measures in an effort to combat corruption and reach global health goals, such as Sustainable Development.
